# Study of the Effect of Selective Media with High Doses of Zinc on Regeneration Ability and Rutin Accumulation in Common Buckwheat In Vitro

**DOI:** 10.3390/plants11030264

**Published:** 2022-01-19

**Authors:** Svetlana Borovaya, Alexey Klykov, Elena Barsukova, Elena Chaikina

**Affiliations:** 1 Federal Scientific Center of Agricultural Biotechnology of the Far East Named after A.K. Chaiki, 30 Volozhenina St., Timiryazevsky Stl., 692539 Ussuriysk, Russia; alex.klykov@mail.ru (A.K.); enbar9@yandex.ru (E.B.); 2 G.B. Elyakov Pacific Institute of Bioorganic Chemistry, Far Eastern Branch of the Russian Academy of Sciences, 690022 Vladivostok, Russia; chaykin.dima@yandex.ru

**Keywords:** *Fagopyrum esculentum*, selective media, zinc, regenerant, morphological indicators, stress resistance, in vitro

## Abstract

Biotechnological methods are widely used in modern common buckwheat (*Fagopyrum esculentum* Moench) studies, constitute an effective tool to create the best agronomic traits of the crop, and can also be used to breed forms, resistant to heavy metal ion toxicity, which is important in the environment of constantly growing anthropogenic pressure on ecosystems. The studied high concentrations of zinc salts (808–1313 mg L^−1^) in the nutrient medium in vitro had an inhibitory effect on buckwheat, which was manifested by a decrease in values of its morphological indicators. Ion stress had an adverse effect on 7–9% of plants from their total number, indicating high plasticity and resistance of *F. esculentum* to highly toxic doses of zinc. The stress state of *F. esculentum* significantly increases the production of flavonoid compounds, including rutin, in plant cells, which is used in biotechnology to assess and obtain buckwheat forms of high flavonoid induction capacity. The processes of rutin biosynthesis were most intense in test-tube plants of the Izumrud × Inzerskaya hybrid obtained after exposure to high doses of zinc 1010–1212 mg L^−1^. *F. esculentum* genotypes obtained using selective backgrounds with high zinc concentrations are promising biosystems for synthesis of rutin, valuable for pharmacology and medicine.

## 1. Introduction

Plant breeding using heavy metal ions as selective backgrounds in vitro can be an effective method to create breeding material tolerant to abiotic stresses [1,2,3]. Furthermore, stress effects trigger the immune mechanisms of plants inducing an increase in flavonoid biosynthesis [4,5], which have great therapeutic and preventive value for human health and contribute to reduced occurrence of many diseases [6,7,8].

Zinc is one of the most important essential trace elements for plants; at the same time, it is a toxic heavy metal that causes oxidative stress and has an adverse effect on the genetic apparatus [9,10,11]. Using cell-tissue breeding, plant forms tolerant to one or more heavy metals have been obtained, resulting in the identification of genotypes with complex resistance to stressors. For example, regenerant plants of bentgrass *Agrostis stolonifera* tolerant to cadmium, lead, copper, and zinc were created using heavy metal ions [12,13,14], in vitro zinc-resistant starting material for *Brassica campestris* and *Brassica juncea* [15], *Setaria* *italis* [16], and *Nicotiana tabacum* [17]. Selective media with elevated concentrations of zinc (46–606 mg L^−1^) and copper (6–230 mg L^−1^) were previously used for cell, tissue and organ culture in buckwheat [2,3,18,19].

As a producer of secondary metabolites, including the use of stress factors, the cell-tissue culture of buckwheat in vitro is quite widely used [20,21,22,23]. A group of Chinese scientists created a highly flavonoid-producing red callus line of *F. cymosum*, which was used for molecular cloning of isoflavone reductase-like gene FcIRL [24]. According to L. Tumova et al. [25], T. Murashige and F. Skoog [26] a mineral-base nutrient medium (MS) with a combination of growth regulators 2,4-dichlorophenoxyacetic acid (1 mg L^−1^) and kinetin (1 mg L^−1^) under normal light conditions is optimal for both in vitro cultivation of *F. esculentum* and flavonoid production. In our studies, it was found that buckwheat callus of some cultivars accumulated anthocyanins during in vitro growth on zinc-containing medium, acquiring pink and crimson coloration [27].

Given the high plasticity and high genetic potential of buckwheat species variability and the lack of information in the literature on experiments with heavy metals on buckwheat in vitro, we aimed to study the effect of selective media containing high doses of zinc on survival, growth and development of buckwheat in vitro, as well as to evaluate their effect on the rutin content in plants, regenerated on nutrient media in test-tubes.

## 2. Results and Discussion

**Figure 1:**
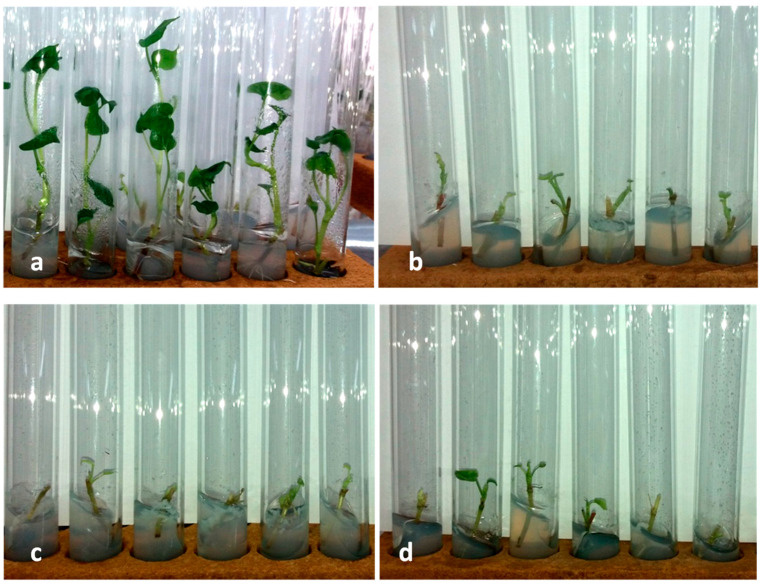
Effect of selective media with zinc on morphological indicators of buckwheat test tube regenerants of Izumrud (21-day cultivation): (**a**) control; (**b**) medium containing 808 mg L^−1^ ZnSO_4_ × 7H_2_O; (**c**) medium containing 1111 mg L^−1^ ZnSO_4_ × 7H_2_O; (**d**) medium containing 1313 mg L^−1^ ZnSO_4_ × 7H_2_O.

It is known that an excess of heavy metal ions leads to oxidative stress in plants and formation of harmful reactive oxygen species, causing a toxic effect and growth retardation [28,29]. Evaluation of development of microclones (one-node cuttings) of *F. esculentum* placed in selective conditions revealed that with increasing concentration of Zn^2+^ ions in nutrient medium, their inhibitory effect on buckwheat increased in all experimental variants, which was manifested in reduction of morphological indicators in comparison with control (Figure 1, Table 1).

Zinc toxicity resulted in a decrease in plant height, number of internodes, leaves, and leaf plate length. Induction of root formation was completely absent. The toxicity of zinc with respect to the morphological characteristics of plant organisms is discussed in detail in a review article by G.R. Rout, P. Das [30], and in research papers by S.D. Pande et al. [31], V. Agrawal and K. Sharma [32], A.F. Titov et al. [33]. Plants, cultivated on selective media acquired green-yellow or brown coloration, indicating the suppression of photosynthesis processes of plants. An adverse effect of heavy metals on photosynthesis in plants has been noted by many researchers [34,35,36]. A decrease in the intensity of photosynthesis of *Rauvolfia serpentina* when exposed to elevated doses of zinc salt in vitro was indicated in the work of N. Ahmad et al. [37].

On the 21st day of cultivation, the most inhibiting effect of Zn^2+^ ions on growth and development of buckwheat Izumrud regenerants was observed on selective media containing 1111–1313 mg L^−1^ of zinc salt, especially at plant height (0.16–0.26 cm on average), number of leaves (1.88–2.25 units), and leaf blade length (2.50–3.38 mm). Regenerants from Izumrud × Inzerskaya hybrid had the lowest plant height (0.11 cm in average), number of internodes (1.00 pieces in average), number of leaves (1.43 pieces in average) and leaf blade length (2.43 mm in average) compared with other variants were recorded at the highest concentration of zinc in nutrient medium 1313 mg L^−^^1^. For other variants, the indicators are slightly higher and do not differ significantly between each other.

Further cultivation of regenerants on MS medium for 33 days revealed that the toxic effects of heavy metal persisted, the plants significantly lagged behind the control group in growth and development (Table 2).

No root formation was observed in regenerants. However, on the variants after the addition of 808–1111 mg L^−1^ of zinc salt, the morphological indicators of buckwheat were significantly higher than on the variants with maximum toxic load, especially in terms of plant height. Thus, whereas in the first case, plant height decreased with increasing zinc concentration in media and varied within 2.98–11.46 cm, in variants following 1212–1313 mg L^−1^ of zinc salts, it was 0.20–0.33 cm, i.e., more than 90% lower. It should be noted that the intensity of regenerants development on media after the toxicant addition at a concentration of 808–1111 mg L^−1^ is much higher than that of the control, as evidenced by the data on their staged development on selective and conventional MS nutrient medium.

As a result, ionic stress with high doses of zinc (808–1313 mg L^−1^) has an adverse effect on 7% of plants of Izumrud variety. Hybrid Izumrud × Inzerskaya proved to be less resistant to the stress factor, viability was 91% of regenerants. It should be emphasized that the study of other crops by different authors revealed a much lower tolerance of plants to zinc stress in vitro. Thus, for *Verbascum phrygium*, the zinc salt content of 250 mg L^−1^ in nutrient medium proved to be critical with a short period of plant exposure equal to 7 days [38]. Significant decrease in shoots growth derived from subcultures on media with ZnSO_4_ × 7H_2_O concentration of 0.48 mM was observed in *Ailanthus altissima* [39]. Adverse effects of low zinc concentrations in in vitro culture were observed in *Bacopa monnieri* [40], *Holarrhena antidysenterica* [31], and aspen (*Populus tremula* × *tremuloides*) [41]. Therefore, based on our data, we can assume that buckwheat is a highly plastic and resistant to long-term stress of highly toxic doses of zinc, as evidenced by the high percentage of surviving plants of *F. esculentum*.

**Figure 2:**
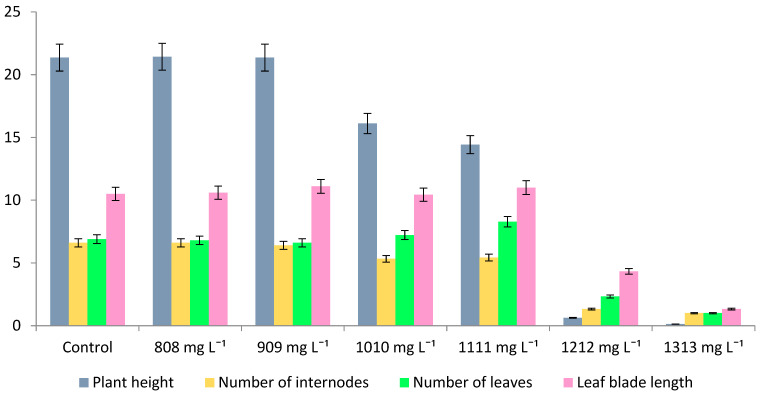
Morphological indicators of buckwheat plants of Izumrud variety on the MS (II passage) after selective media with Zn^2+^ on the 33rd day of cultivation.

**Figure 3:**
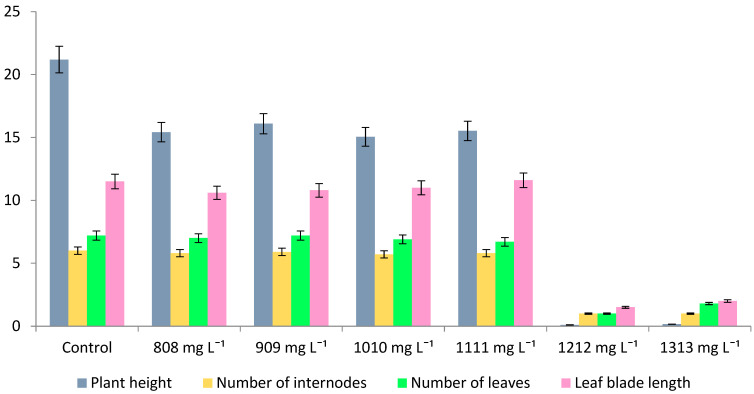
Morphological indicators of plants of buckwheat hybrid Izumrud × Inzerskaya on the MS (II passage) after selective media with Zn^2+^ on the 33rd day of cultivation.

Subsequent passage (II) on MS nutrient media let the surviving buckwheat lines from the variants 808–1111 mg L^−1^ recover and show high rate of growth and development their morphological indicators turned out to be similar to those of the control variant or slightly lower (Figure 2 and Figure 3). Rhizogenesis, the most important indicator characterizing the adaptive capabilities of plants, was observed on all plant variants, except those with 1212–1313 mg L^−1^ of zinc salt. 

The pattern observed in the third passage (III) on the MS was similar to the II passage.

**Figure 4:**
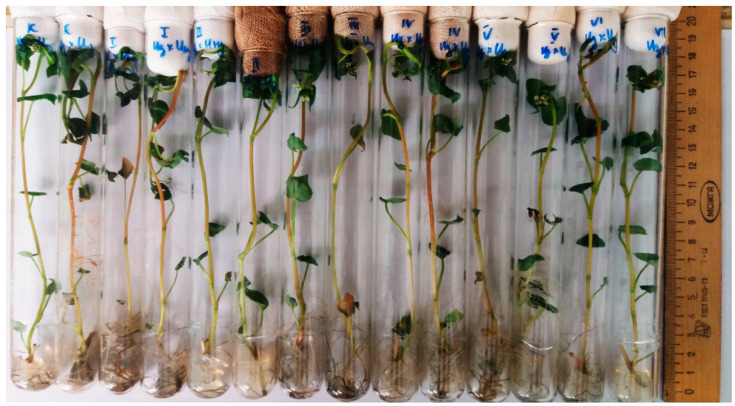
Buckwheat regenerants (hybrid Izumrud × Inzerskaya, variants following treatment with 808–1313 mg L^−1^ of zinc salt), IV passages on the MS.

Buckwheat plants in the variants with zinc salt concentration of 1212–1313 mg L^−1^ restored their ability to root formation after subsequent microcloning in growth media MS (Figure 4). All morphological indicators of plants, such as their height, number of internodes, number of leaves, length of leaf blade, leaf color, were similar to those of the control variant.

The revealed ability of the studied genotypes to grow under extreme stress conditions shows a high degree of plasticity of *F. esculentum*. This feature of buckwheat is emphasized by I. Kreft [42]. Tolerance of buckwheat microstems to elevated concentrations of copper and zinc sulfate in vitro was mentioned earlier [2]. According to M. Germ and A. Gaberščik [43], different species and varieties of buckwheat, originating from relatively harsh habitats [44], can be classified as the most stress-resistant crops.

**Figure 5:**
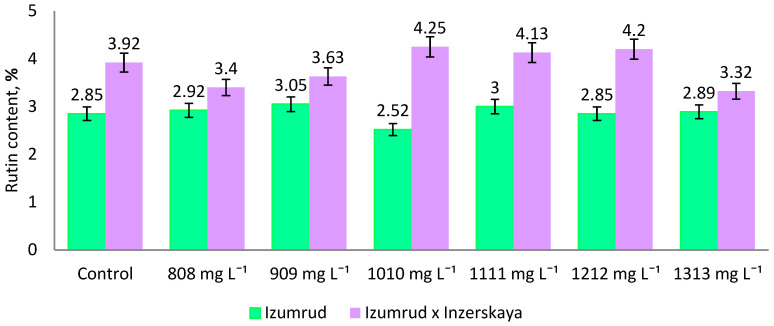
Rutin content in *F. esculentum* regenerants cultured in vitro during III-V passages on the MS after exposure to selective media with zinc.

The results of the study of the flavonoid compound rutin content in in vitro cultivated test tube plants of *F. esculentum* obtained after zinc exposure are shown in Figure 5. We were interested to find out whether the studied genotypes, which were exposed to selective media with high doses of zinc, could retain the ability to perform biosynthesis of flavonoid compounds during the subsequent stages of microclonal propagation, especially since there are no such data the available literature. Therefore, plant material for determination of rutin was collected during III-V subsequent passages of regenerants obtained on MS nutrient media.

The plants, which are capable of adapting to stress factors, including the effects of heavy metals, are known to have the content of phenolic compounds, including flavonoids, in cells, which is reported to be significantly higher than those of the forms characterized by low viability and adaptive response [45,46]. In our studies, the highest rutin content (3.32–4.25%, average value 3.84%) was found in the hybrid Izumrud × Inzerskaya, which is 1.34 times as hight as that in the variety Izumrud (2.52–3.05%, average value 2.86%). The maximum amount of rutin, significantly exceeding the control, was recorded in the hybrid Izumrud × Inzerskaya in the variants after 1010–1212 mg L^−1^ of zinc salt 4.13–4.25% of rutin. It is interesting to note that the minimum accumulation of flavonoid was observed after exposure to the highest test concentration of 1313 mg L^−1^ zinc salt, which was 3.32%, which is 1.2 times as low as that in the control group. An increase of flavonoid rutin biosynthesis in buckwheat (1.25-time higher compared with the control group) under the effect of elevated zinc salt concentration (404 mg L^−1^) in vitro was demonstrated in our earlier works [2].

Increased formation of phenolic compounds in plants under metal stress is a protective response to oxidative stress and plays an important role in cell protection, adaptation and ability to survive under adverse conditions [47,48,49,50,51]. The studies of K. Ikram et al. [52] found an increase in the content of polyphenolic compounds in the aboveground and root parts of *Atriplex canescens* when the concentration of zinc in the nutrient substrate increased. J.E.J. Keziah et al. [53] showed that flavonoid content increased in suspension culture of *Indigofera tinctoria* when exposed to lead. Similar data were obtained when studying the effect of cadmium on callus cultures of long-stem flax [54] and tea plant [55]. However, the Izumrud variety did not differ significantly from the control in terms of rutin content. The different response of genotypes to stress conditions is expected, since the level of resistance to stress and, therefore, to the accumulation of flavonoid rutin, is a genetically controlled and inherited trait. It is believed that the main mechanism of adaptation to metal stress is the activation of the biosynthesis of key biosynthetic enzymes including chalconoisomerase (CHI), phenylalanine ammonia-lyase (PAL), cinnamate-4-hydroxylase (C4H), 4-coumarate-CoA ligase (4CL) and others [56,57], due to the elevated levels of corresponding encoding gene transcripts [58,59]. Thus, our data indicate that, in contrast to Izumrud variety, the increased level of expression of rutin biosynthesis genes is maintained during subsequent microcloning in plants of Izumrud × Inzerskaya hybrid exposed to high doses of zinc salt of 1010–1212 mg L^−1^.

## 3. Materials and Methods

### 3.1. Original Plant Material

In this study, seeds of buckwheat variety Izumrud and genetically aligned hybrid population (generation F_6_) Izumrud × Inzerskaya selection of A.K. Chaiki Federal Schientific Centre for Agrobiotechnology of the Far East, Primorsky Territory, Russia, were used as primary explants.

### 3.2. Introduction into In Vitro Culture and Obtaining Regenerant Buckwheat Plants

To introduce mature buckwheat seeds into the culture, they were sterilized according to the method of V.A. Tilba [60] as follows: seeds were immersed for 2 min in concentrated sulfuric acid, then washed 3 times 5 min each with autoclaved distilled water, then freed from pericarp in sterile conditions of the box. Primary explants were passaged on hormone-free nutrient medium with a mineral base according to Murashige-Skoog [26], containing 20 g L^−1^ of sucrose, 6 g L^−1^ of agar, and 1 g L^−1^ of casein hydrolysate. Isolated in vitro objects were cultured in tubes with cotton-gauze plugs at 4000-lx illumination, 22–25 °C temperature, and 16-h u photoperiod under culture room conditions. Buckwheat in vitro regenerant plants were obtained during multiple passages. Duration of one passage was 30 days. Microcloning of buckwheat plants was performed according to the method developed at FSC of Agricultural Biotechnology of the Far East named after A.K. Chaika [61], which included splitting a shoot (2–3 lower internodes) into 15–20 mm long cuttings with axillary buds and their cultivation on hormone-free MS nutrient medium containing sucrose, agar and casein hydrolysate as described above. Box, dishes, and instruments were prepared and sterilized according to generally accepted methods.

### 3.3. Composition of Selective Media and Experiments

To create selective conditions, zinc salt (ZnSO_4_ × 7H_2_O) was added to MS nutrient medium in the following amounts according to the experiment variants: 808, 909, 1010, 1111, 1212, and 1313 mg L^−1^. Aseptic single node cuttings, were cultured for 33 days on MS medium with standard content (8.6 mg L^−1^) of zinc sulfate (control) and selective media with zinc according to the experiment variants. The number of test tubes for each variant was 20; the number of repetitions was 5. The surviving genotypes were microcloned onto MS nutrient media. The duration of each passage on medium was 33 days.

### 3.4. Determination of Morphological Indicators of Regenerant Plants

Morphological indicators were determined on the 21st day of cultivation of plants on control and selective media with zinc, as well as at the end of subsequent passages on MS nutrient media. The height of each test-tube plant and the length of its leaf plates were measured and expressed, respectively, in cm and mm, the number of internodes and the number of leaves (pcs) were counted, and the presence (+) or absence (−) of roots was noted. Leaf color was determined visually by the predominant color of all leaf blades of the plant.

### 3.5. Determination of Rutin Content

Rutin content was determined in buckwheat regenerants (without roots) cultured in vitro on conventional MS nutrient media for III-V passages, obtained after exposure to selective media with zinc. Rutin extraction was performed according to the method I. Kreft et al. [62]. To identify rutin, we used NMR ^1^H spectra, which were recorded on a Bruker AC 250 MHz spectrometer (Bruker BioSpin Gmbh, Germany) in CDCl_3_ (deuterochloroform) and acetone-d6, and compared with individually pure rutin (Chemopol, Czech Republic). Mass spectra were obtained on a chromatograph-mass spectrometer LKB-9000 (LKB INSTRUMENTS, INC, Sweden) with direct input at ionizing electron energies of 18 and 70 eV. The amount of rutin was determined by the chromatospectrophotometric method developed by G.I. Vysochina [63].

### 3.6. Statistical Analysis

Microsoft Excel 2010 software package was used for data entry, initial data processing, and statistical analysis. Statistica 6 [64] software was used to perform single-factor analysis of variance. Duncan’s multiple range test (*p* < 0.05) was used to measure the significance of differences. Results are expressed as mean values ± standard deviation.

## 4. Conclusions

High concentrations of zinc salts (808–1313 mg L^−1^) in the nutrient medium in vitro have an inhibitory effect on plants of buckwheat cultivar Izumrud and hybrid Izumrud × Inzerskaya, which is expressed in the reduction of morphological indicators and suppression of rhizogenesis. Buckwheat is a crop which is very plastic and resistant to prolonged stress exposure to highly toxic doses of zinc. The processes of biosynthesis of flavonoid compound rutin were most intensive in test-tube plants of Izumrud × Inzerskaya hybrid obtained after high doses of zinc exposure (1010–1212 mg L^−1^). The highest rutin content was found in hybrid Izumrud × Inzerskaya, 1.34 times higher than in cultivar Izumrud, which is probably due to properties of the genotype of the studied samples. Cell-tissue cultures of *F. esculentum* obtained using selective backgrounds with high doses of zinc are promising biosystems for the synthesis of the flavonoid compound rutin, valuable for pharmacology and medicine. This work describes only a laboratory study of the effect of zinc stress on the development of buckwheat plants and their ability to increase rutin biosynthesis in vitro. Field studies are neddedto obtaine regenerant plants and their seed progeny.

## Figures and Tables

**Table 1:** Effect of selective media with ZnSO_4_ × 7H_2_O on morphological indicators of buckwheat regenerants at 21 days of cultivation.

ZnSO_4_ × 7H_2_O Treatment	Plant Height (cm)	Number of Internodes (pcs.)	Number of Leaves (pcs.)	Leaf Blade Length (mm)	Roots (+/−)	Leaf Color
Izumrud						
Control 8.6 mg L^−1^	5.98 ± 4.70 b	3.20 ± 0.60 b	5.10 ± 0.30 c	14.40 ± 2.20 d	+	green
808 mg L^−1^	0.86 ± 0.32 a	1.43 ± 0.79 a	2.86 ± 0.90 b	3.57 ± 0.79 ac	−	green and yellow
909 mg L^−1^	0.64 ± 0.30 a	1.22 ± 0.44 a	2.78 ± 0.67 b	5.22 ± 2.17 c	−	yellow
1010 mg L^−1^	0.43 ± 0.24 a	1.00 ± 0.00 a	2.40 ± 0.97 ab	7.50 ± 3.31 b	−	yellow
1111 mg L^−1^	0.16 ± 0.07 a	1.00 ± 0.00 a	2.13 ± 1.13 a	3.38 ± 1.68 ac	−	yellow
1212 mg L^−1^	0.21 ± 0.08 a	1.00 ± 0.00 a	1.88 ± 1.13 a	2.71 ± 0.95 a	−	brown and yellow
1313 mg L^−1^	0.26 ± 0.17 a	1.00 ± 0.00 a	2.25 ± 1.16 ab	2.50 ± 1.20 a	−	brown and yellow
**Izumrud × Inzerskaya**						
Control 8.6 mg L^−1^	5.43 ± 3.77 b	3.63 ± 0.52 b	5.38 ± 0.52 c	13.63 ± 4.34 c	+	green
808 mg L^−1^	0.53 ± 0.28 a	1.11 ± 0.33 a	3.11 ± 0.60 b	5.56 ± 1.24 ab	− −	green and yellow
909 mg L^−1^	0.51 ± 0.37 a	1.14 ± 0.38 a	2.86 ± 1.07 b	4.86 ± 1.78 ab	−	green and yellow
1010 mg L^−1^	0.49 ± 0.57 a	1.20 ± 0.42 a	2.20 ± 0.92 a	5.10 ± 2.08 ab	−	yellow
1111 mg L^−1^	0.77 ± 0.82 a	1.17 ± 0.41 a	2.50 ± 0.55 ab	6.50 ± 3.27 b	−	yellow
1212 mg L^−1^	0.46 ± 0.61 a	1.20 ± 0.45 a	2.20 ± 0.84 a	3.60 ± 1.34 ab	−	brown
1313 mg L^−1^	0.11 ± 0.04 a	1.00 ± 0.00 a	1.43 ± 0.53 a	2.43 ± 0.53 a	−	brown

The data in the table are represented as the mean ± standard deviation, and different lowercase letters in the same column indicate significant differences between treatments at *p* < 0.05.

**Table 2:** Morphological indicators of buckwheat regenerants on MS medium after addition of zinc.

ZnSO_4_ × 7H_2_O Treatment	Plant Height (cm)	Number of Internodes (pcs.)	Number of Leaves (pcs.)	Leaf Blade Length (mm)	Roots (+/−)	Leaf Color
Izumrud						
Control 8.6 mg L^−1^	18.90 ± 2.41 c	6.00 ± 0.67 cd	6.70 ± 0.67 c	14.80 ± 2.50 c	+	green
808 mg L^−1^	8.71 ± 4.64 b	5.86 ± 3.63 c	11.43 ± 4.89 f	6.71 ± 2.81 b	−	green and yellow
909 mg L^−1^	5.68 ± 4.58 b	4.10 ± 1.85 bc	7.60 ± 3.37 cd	7.60 ± 3.24 b	− −	green and yellow
1010 mg L^−1^	4.45 ± 4.58 b	3.25 ± 2.63 b	4.00 ± 3.16 bcd	4.50 ± 3.00 ab	−	yellow and green
1111 mg L^−1^	4.35 ± 2.62 b	4.00 ± 1.41 bc	8.50 ± 3.54 cf	6.50 ± 2.12 b	−	yellow and green
1212 mg L^−1^	0.33 ± 0.15 a	1.00 ± 0.00 a	2.00 ± 1.00 b	2.67 ± 0.58 a	−	brown and yellow
1313 mg L^−1^	0.20 ± 0.00 a	1.00 ± 0.00 a	1.00 ± 0.00 a	3.00 ± 0.00 a	−	brown and yellow
**Izumrud × Inzerskaya**						
Control 8.6 mg L^−1^	17.61 ± 4.60 b	5.44 ± 0.73 b	6.78 ± 1.30 bc	14.56 ± 4.44 c	+ +	green
808 mg L^−1^	11.46 ± 3.85 b	5.86 ± 0.90 b	11.43 ± 5.19 c	8.14 ± 1.57 b	− −	green and yellow
909 mg L^−1^	6.90 ± 6.47 b	4.20 ± 3.27 b	7.60 ± 5.32 c	7.40 ± 3.29 b	− −	green and yellow
1010 mg L^−1^	5.65 ± 6.75 a	3.50 ± 2.39 ab	5.88 ± 3.56 abc	6.75 ± 2.92 b	− −	yellow and green
1111 mg L^−1^	2.98 ± 3.66 a	2.80 ± 2.05 ab	4.60 ± 2.41 abc	8.00 ± 5.05 b	− −	yellow and green
1212 mg L^−1^	0.23 ± 0.06 a	1.00 ± 0.00 a	1.67 ± 0.58 ab	2.00 ± 0.00 a	− −	brown and yellow
1313 mg L^−1^	0.30 ± 0.20 a	1.00 ± 0.00 a	1.00 ± 1.00 a	1.67 ± 1.53 a	− −	brown and yellow

The data in the table are represented as the mean ± standard deviation, and different lowercase letters in the same column indicate significant differences between treatments at *p* < 0.05.

## Data Availability

All data are included in this study.
